# Impact of qualitative Diffusion weighted imaging and apparent diffusion coefficient (DWI/ADC) on target volume delineation in cervical cancer: a feasibility study

**DOI:** 10.1016/j.tipsro.2026.100424

**Published:** 2026-08-13

**Authors:** Ankita Mehta, Adarsh Ishwar Hegde, Jayashree N P, Umesh Velu, Shirley Lewis

**Affiliations:** a Department of Radiation Oncology, Kasturba Medical College, Manipal Academy of Higher Education, Manipal, India; b Department of Radiodiagnosis and Imaging, Kasturba Medical College, Manipal Academy of Higher Education, Manipal, India

**Keywords:** Magnetic resonance imaging, Target delineation, Image guided adaptive brachytherapy, Functional MRI, Diffusion weighted imaging, Apparent diffusion coefficient

## Abstract

**Background:**

Magnetic resonance imaging (MRI) based IGABT in cervical cancers relies on differential target volume delineation on T2-weighted imaging. Despite decades of experience, there are certain inconsistencies in delineation, with the gross tumor volume (GTV) demonstrating the least concordance. Analyzing current imaging methodologies, in the context of functional imaging, can possibly conceptualize GTV delineation.

**Methods:**

MRI pelvis datasets of 20 cervical cancer patients at pre-external beam radiotherapy (EBRT) and pre-brachytherapy were selected. The GTV was delineated on the T2 sequence alone and on the fused T2/DWI/ADC sequences. Quantitative and spatial differences between the two delineation methods were analyzed.

**Results:**

At pre-EBRT, the median absolute difference between GTV_T2 and GTV_T2/DWI was 2 cm^3^ (−1.72; 8.27, *p* = 0.117), with strong positive correlation (ρ = 0.943) and moderate to good spatial agreement (Volume ratio: 0.90, DICE Similarity Coefficient (DSC): 0.75, Jaccard Index (JI): 0.61). All lateral tumor extent measurements on one side were higher on T2 imaging, with a maximum of 2.63 cm (1.83;2.87) versus 2.05 cm (1.78;2.39) (*p* < 0.0001). At pre-brachytherapy, the median difference was 0.30 cm^3^ (−0.07; 0.77, *p* = 0.017), with moderately strong correlation (ρ = 0.707) and low to moderate spatial agreement (Volume ratio: 0.79, DSC: 0.33, JI: 0.19). The lateral tumor extent measurement at one of the levels was significantly higher on T2 imaging, being 0.95 cm (0.60;1.78) versus 0.81 cm (0.00;1.39) (*p* = 0.033).

**Conclusion:**

The two volumes showed strong correlation; however, small yet statistically significant differences were observed at the pre-brachytherapy, with lesser spatial agreement. T2-only imaging demonstrated higher lateral tumor extent at both time points. Overall, these findings indicate discernible differences with functional imaging.

## Highlights

• Integrating functional imaging with standard T2-weighted imaging for tumour delineation can depict a spatially distinct disease map.
• The spatial agreement between functional imaging–defined volumes and T2-weighted volumes was relatively higher at the pre-EBRT compared to the pre-brachytherapy.
• These differences at pre-brachytherapy may have important implications for brachytherapy given the steep dose gradients and differential dose–defining volumes.

## Background

T2-weighted Magnetic resonance imaging (MRI) plays a central role in image-guided adaptive brachytherapy (IGABT) for cervical cancer, where baseline and pre-brachytherapy tumor extent define the target volumes [1]. Improved local control has shifted failure patterns toward distant relapse [2], [3]. Patterns of local relapse depict predominance of in-field failures [4], and the delineation of gross tumor volume (GTV) is not well corroborated [5], depicting the need to further refine the imaging and delineation methodologies. Functional MRI, particularly diffusion-weighted imaging (DWI) and apparent diffusion coefficient (ADC) maps, offers improved visualization of viable tumor due to inherent ability to detect physiological changes [6], [7], [8]. Although widely used in other malignancies [[9], [10], [11], [12], [13], [14]], its role in cervical cancer remains investigational [15], [16] despite demonstrated diagnostic and response-assessment utility [17]. This study evaluates the feasibility of incorporating DWI/ADC for GTV delineation by comparing tumor volume metrics with standard T2-weighted imaging.

## Methods

### Patient selection

The design and conduct of this study was reviewed and approved by the Institutional Ethics Committee of Kasturba Medical College and Kasturba Hospital (IEC_241/2025). All procedures involving human participants were performed in accordance with the ethical standards laid down in the 1964 Declaration of Helsinki and its later amendments. The study is a retrospective analysis of diagnostic MRI datasets, and no additional interventions or patient contact were undertaken for research purposes. The datasets were de-identified for data collection and patient confidentiality was maintained. A waiver of informed consent was granted by the Institutional Ethics Committee.

The diagnostic imaging of cervical cancer patients treated by definitive chemoradiotherapy from June 2023 till February 2025 was screened. Patients with a complete MR pelvis dataset (inclusive of T2, DWI, ADC) at pre-external beam radiotherapy (EBRT), and pre-brachytherapy were identified. These auto co-registered datasets were imported to Varian Brachytherapy planning system, and automatic fusion between T2 and DWI sequences was verified in the axial, sagittal, and coronal place with cervical canal as the anatomical reference.

Datasets demonstrating a fusion discrepancies of ≤2 mm within the region of interest were selected. A total of 20 patients were deemed suitable, with 40 datasets at two time points. The GTV metrics were compared between T2-weighted imaging alone and in combination with DWI/ADC sequences.

### MRI imaging protocol

MRI pelvis was performed on a 1.5-T Philips scanner using body and pelvic phased-array surface coils. No intravaginal gel, contrast, or applicator was administered prior to scanning. The imaging protocol included T2-weighted turbo spin-echo sequences acquired in supine position in axial, coronal and parasagittal planes, with an in-plane resolution of ≤0.2 cm, slice thickness of 0.5 cm, and no interslice gap. DWI was done using single-shot echo-planar imaging in the axial plane with identical spatial resolution parameters. Diffusion gradients were applied along three orthogonal directions with b-values of 0 and 800 s/mm^2^. ADC maps were generated from the DWI dataset using the scanner console software.

### Previous treatment details

All patients underwent definitive radiotherapy with weekly concurrent chemotherapy with an overall treatment time of seven weeks. Radiation was planned according to the EMBRACE II protocol [18].

#### EBRT

EBRT was planned for a dose of 45 Gy in 25 fractions, with a sequential or simultaneous boost to the involved lymph nodes to a cumulative dose of 55–57.5 Gy. The planning technique used was either 3-Dimensional Conformal Radiotherapy or Volumetric Modulated Arc Therapy.

#### Brachytherapy

Applicator selection was based on tumor topography, using intracavitary, hybrid, or interstitial techniques. Treatment was delivered in four fractions over two applications at an interval of one week, with a dose of 7 Gy per fraction.

### MRI based target delineation methodology

**Fig. 1:**
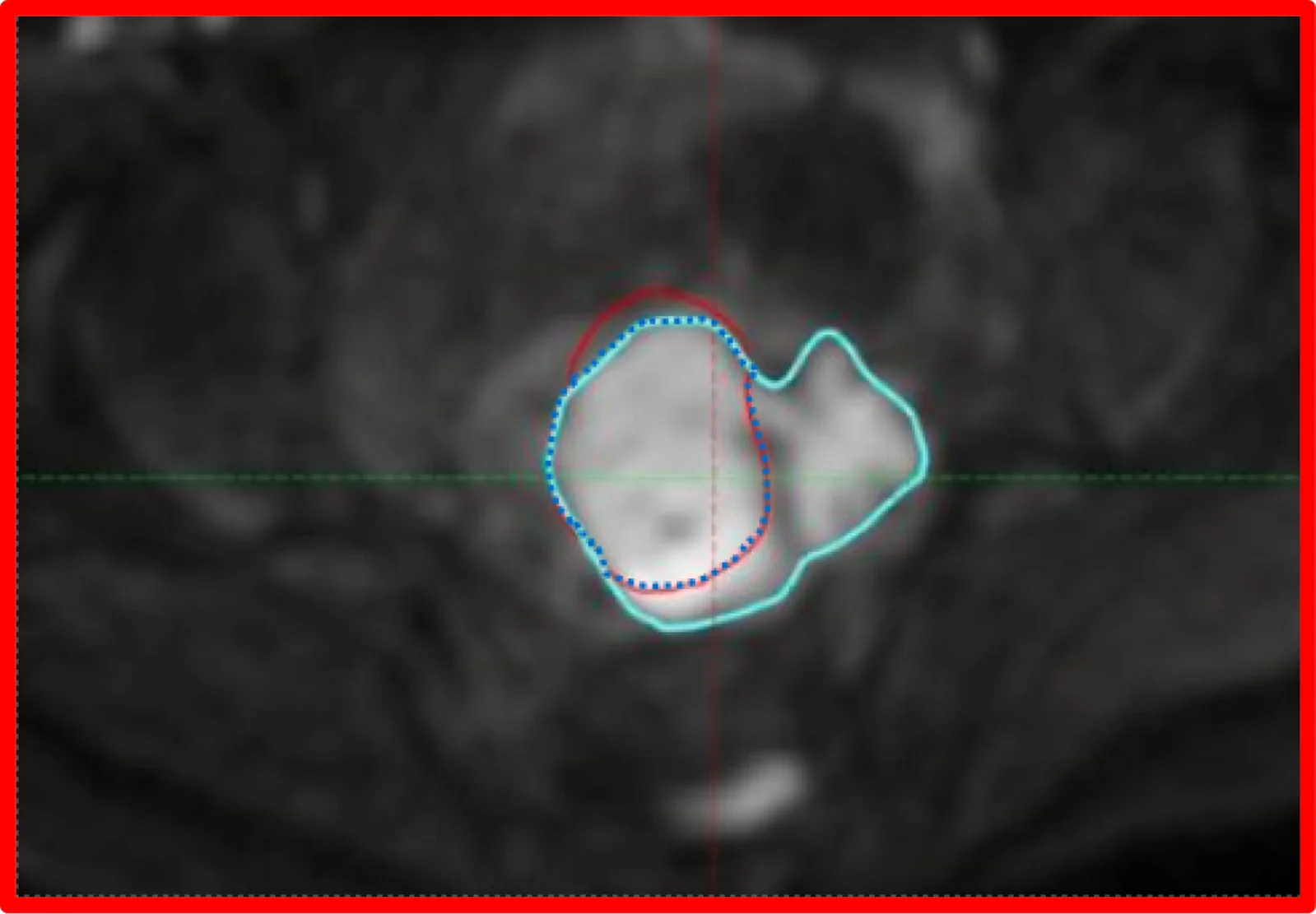

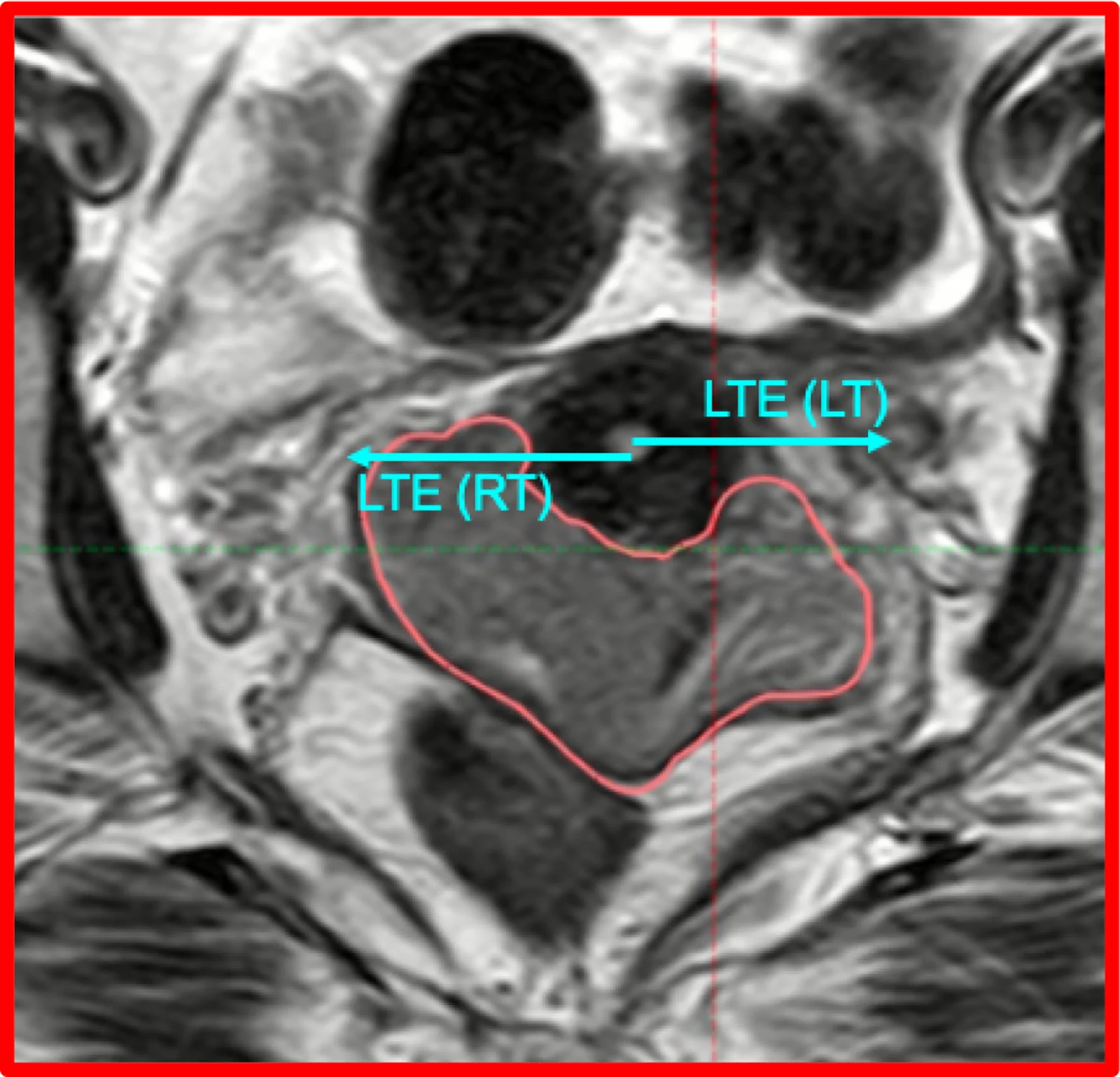
a. Figure depicting GTV_T2 in red outline and GTV_T2/DWI in cyan outline and the overlap volume in dotted blue outline. (For interpretation of the references to colour in this figure legend, the reader is referred to the web version of this article.) b. Figure depicting the measures of Lateral Tumor Extent on axial T2 weighted MRI on right, LTE (RT) and left side, LTE (LT).

The GTV was delineated on the diagnostic pre-EBRT, and pre-brachytherapy datasets and readers were blinded to clinical findings and previous treatment target volumes. The GTV_T2 included hyperintense and intermediate signal regions on T2-weighted imaging, and GTV_T2/DWI incorporated DWI hyperintense regions with corresponding ADC hypointensity, using the T2 sequence for anatomical correlation (Fig. 1). Consensus contours for both volumes were generated sequentially by a radiologist and a radiation oncologist. The methodology was standardized through a joint review with two additional experts for the initial five cases. The high-risk clinical target volume (HRCTV) in the pre-EBRT scans was delineated as the GTV at the time of diagnosis, adjacent tissues with a margin of around 5 mm cropped from the anatomical barriers and the whole cervix while in the pre-brachytherapy scans, it included the residual GTV, and the whole cervix [19]. Additionally, three volumes were generated that included the regions of overlap and combination of GTV_T2 and GTV_T2/DWI and the GTV_T2/DWI beyond the HRCTV.

### Data collection

#### Clinical parameters

Baseline patient, disease, treatment characteristics and clinical outcome data at the last follow-up visit was recorded. The response categories at the time of brachytherapy were classified according to the IBS–ABS–ESTRO guidelines [20].

#### Imaging parameters

The GTV at the baseline and pre-brachytherapy scans were compared in terms of:-

1. **Volumetric parameters**

• Absolute difference = GTV_T2 (cm^3^) – GTV_T2/DWI (cm^3^)
• Percentage difference = [Absolute difference (cm^3^) X 100] / Average volume (cm^3^)
• Correlation coefficient: absolute volumes of GTV_T2 and GTV_T2/DWI (at pre-EBRT and pre-brachytherapy), percentage differences among the two time points, and between the absolute volumes of GTV_T2 and percentage differences

2. **Lateral tumor extent:** Measurements were taken from the center of the uterocervical canal to the lateral-most extent of the GTV on the right and left sides at three axial levels. The cranial level corresponded to the uterine artery, with subsequent measurements taken 1 cm and 2 cm caudal to it. The maximum extent for each side was defined as the largest of the three measurements (Fig. 1b).
3. **Spatial overlap indices:**

• DICE Similarity Coefficient (DSC) = (2 X Overlap Volume)/ Boolean Volume.
• Jaccard Index (JI) = Overlap volume/ (Boolean volume – Overlap volume).
• Volume ratio = Absolute GTV_DWI volume/ Absolute GTV_T2 volume.
• Mean Hausdorff Distance (MHD) = average of the minimum distances between the maximum lateral tumor extent, on right and left sides.

In pre-brachytherapy imaging, for cases with no volumes for both GTV_T2 and GTV_DWI volumes, the DSC, JI, and volume ratio were considered as 1 and MHD as 0.

### Statistical analysis

Statistical analyses were performed using SPSS version 25. Descriptive statistics were used to summarize patient characteristics and volume parameters. Data distribution was assessed using the Shapiro–Wilk test. The Wilcoxon signed-rank test was used for paired comparisons, the Mann–Whitney *U* test for exploratory analyses, and Spearman's rank correlation test for correlation analyses. A *p*-value <0.05 was considered statistically significant.

## Results

### Patient characteristics

Of the 20 patients were studied, 19 of them had cervical cancer, and 1 had vault cancer. The median population age was 55 years. (IQR: 49; 58 years). The study cohort largely comprised 95% patients with locally advanced disease, and 50% of the patients had either pelvic or para-aortic lymph nodal involvement. The predominant histology was Squamous Cell Carcinoma found in 85% patients. At brachytherapy, 60% had response category 1 with either no residual disease or residual disease confined only to cervix, 35% had category II with residual disease in proximal parametrium/upper third vagina, and 5% had category III with residual disease in distal parametrium/middle or lower third vagina or distal uterine corpus (Table 1). Over a median follow up of 10.9 months (IQR: 7.9; 21.2), the locoregional control rates were 100% and the distant control rates were 85%.

**Table 1:** Table depicting the baseline patient, tumor and treatment characteristics.

Characteristic	N (%)
***Primary site***	
Cervix	19 (95)
Vault	1 (5)

***Histology***	
Squamous Cell Carcinoma	17 (85)
Adenocarcinoma	2 (10)
Mucinous adenocarcinoma	1 (5)

***Stage***	
II A1	1 (5)
II A2	1 (5)
II B	5 (25)
III B	3 (15)
IIIC1	7 (35)
IIIC2	3 (15)

***Response category***	
I	12 (60)
II	7 (35)
III	1 (5)

***Brachytherapy application type***	
Intracavitary	6 (30)
Intracavitary and interstitial	13 (65)
Interstitial	1 (5)

### Imaging characteristics

#### Pre EBRT comparison

**Volumetric comparison:** The absolute difference between GTV_T2 and GTV_T2/DWI volumes was a median of 2 cm^3^ (IQR: −1.72; 8.27) with respective median absolute values of 35.25 cm^3^ and 23.75 cm^3^ respectively with no statistically significant difference (*p* = 0.117). The percentage difference was a median of 10.25%, ranging from - 62.6% to +44.7%. Of the 20 patients studied, 14 exhibited a smaller volume on the DWI scan, 5 had a larger volume, and 1 had equal volumes. The two volumes demonstrated a strong positive correlation, (ρ = 0.943, *p*-value <0.0001). The lateral tumor extents were significantly lower at all levels on the right side, with the maximum measure being 2.63 cm (1.83; 2.87) for GTV_T2 and 2.05 cm (1,78; 2.39) for GTV_T2/DWI (*p* < 0.0001) while the comparison on the left side showed no statistically significant difference. In the comparison of maximum lateral tumor extent for both right and left sides, 30 values (75%) were within 5 mm, 6 (15%) ranged between 6 and 10 mm, and 4 (10%) were within 11–15 mm. In all patients except two, the GTV_T2/DWI volume was entirely.

**Fig. 2:**
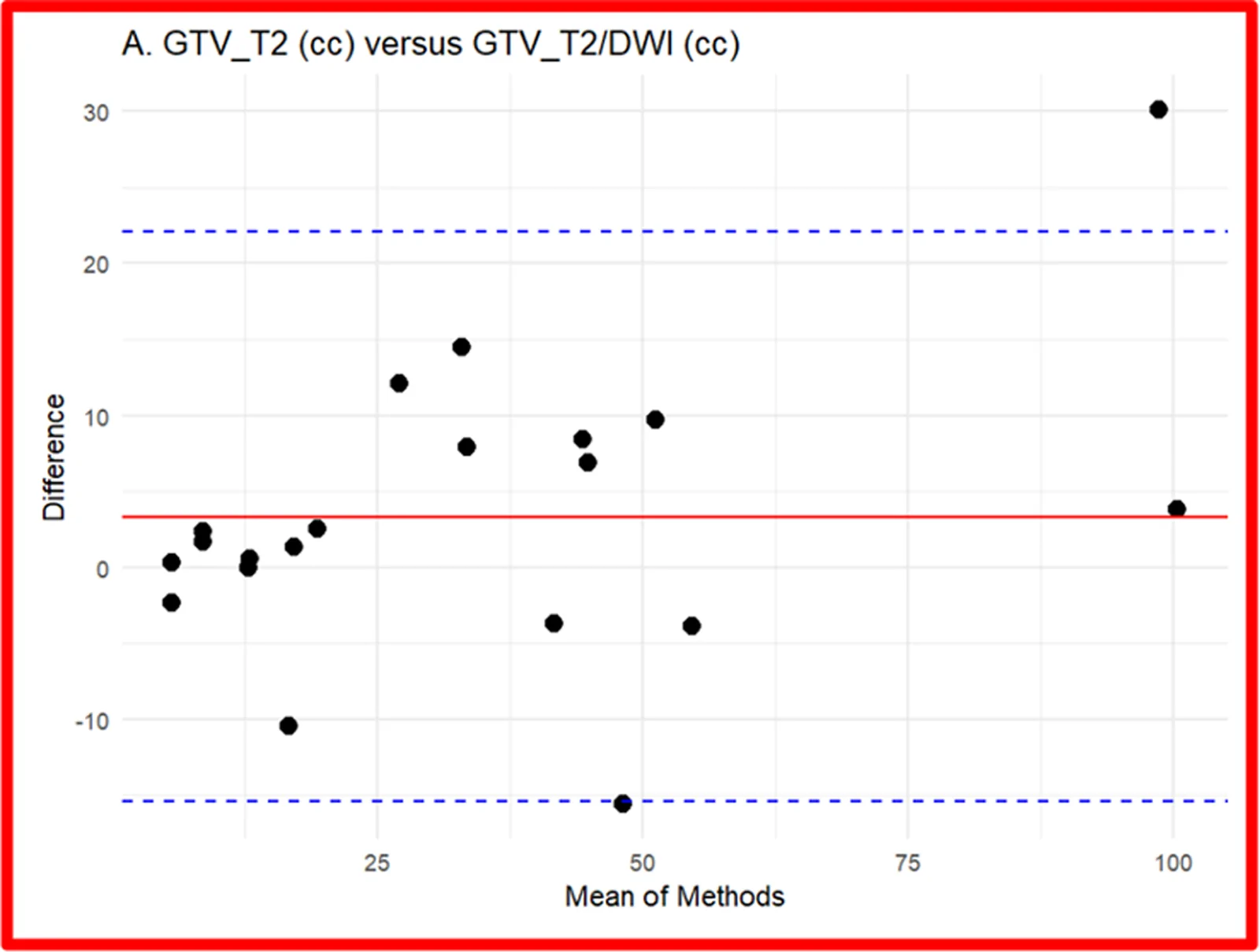

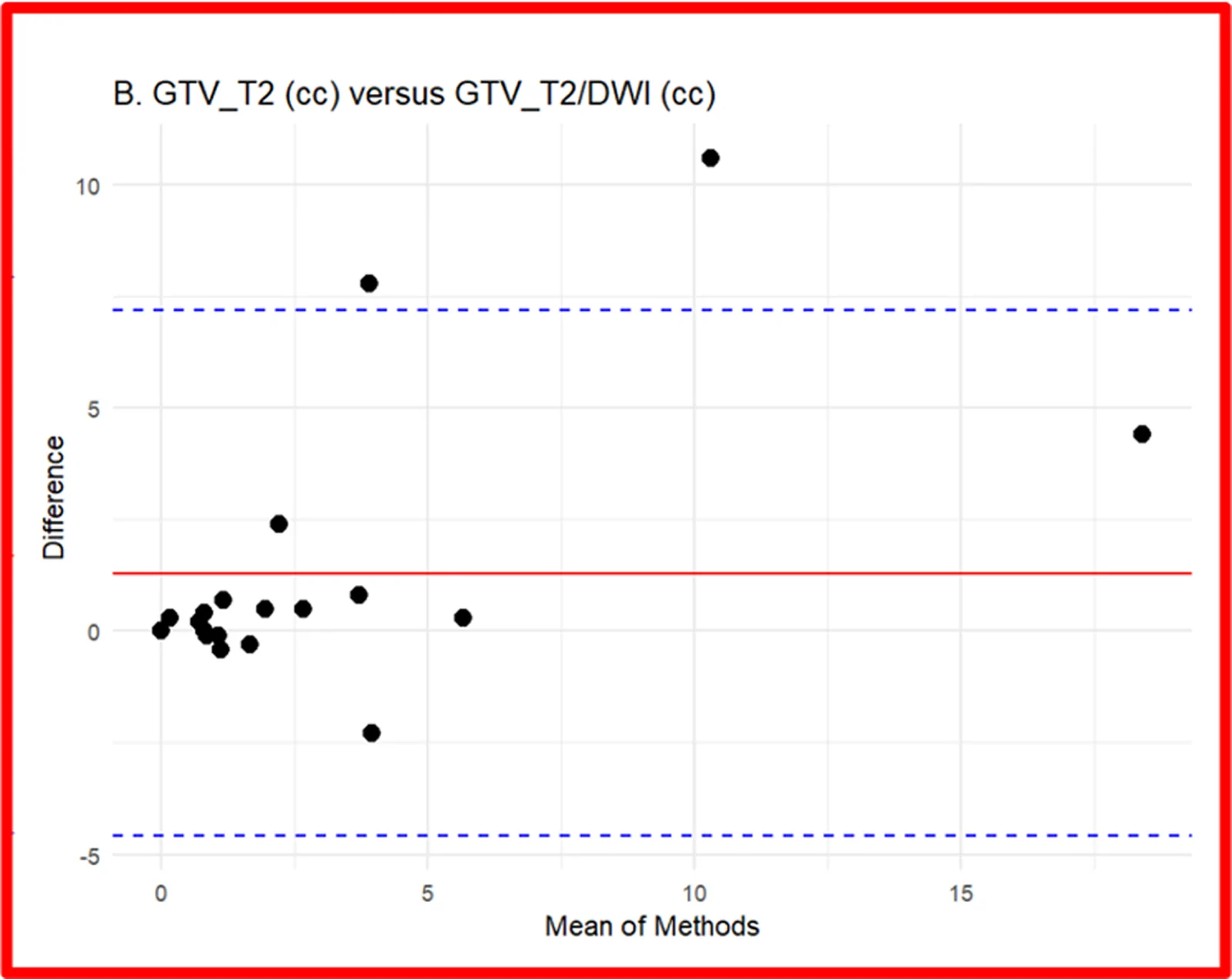

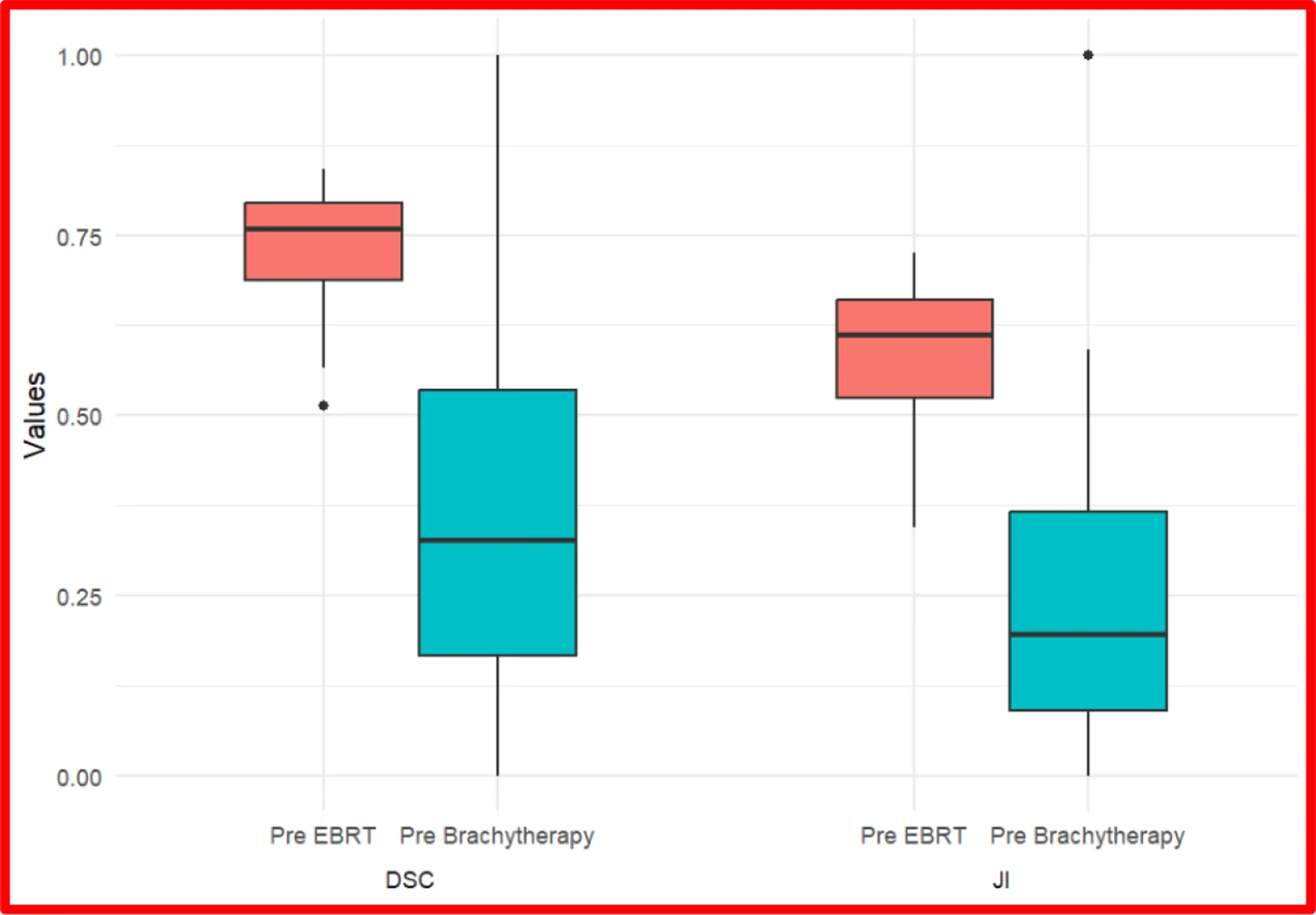
a. Bland–Altman plot illustrating the differences in absolute volumes between GTV_T2 and GTV_T2/DWI at pre-EBRT. b. Bland–Altman plot illustrating the differences in absolute volumes between GTV_T2 and GTV_T2/DWI at pre-brachytherapy. c. Paired Box plot illustrating the overlap indices (DSC & JI) at pre-EBRT and pre-brachytherapy.

Encompassed within the HRCTV, with the volumes in the remaining two cases measuring 0.1 cm^3^ and 1.9 cm^3^ respectively. (Table 2). Also, the absolute tumor volume based on GTV_T2 showed no correlation with the percentage difference (ρ = 0.202; *p* = 0.394). Bland-Altman plots illustrating the differences at pre-EBRT are shown in Fig. 2.

**Table 2:** : Table depicting the pre-EBRT statistical comparison.

Parameter	GTV_T2 Median (IQR)	GTV_T2/DWI Median (IQR)	*P* value
**Absolute volume (**cm^3^**)**	35.25 (11.75;48.42)	23.75 (12.65; 45.60)	0.117
**Right Lateral Tumor Extent (cm)**			
Uterine artery	1.80 (1.60; 2.37)	1.74 (1.27; 2.12)	0.005
1 cm below	2.32 (1.62; 2.78)	1.80 (1.23; 2.44)	0.001
2 cm below	2.30 (0.19; 2.75)	1.90 (0.76; 2.26)	0.026
Maximum	2.63 (1.83; 2.87)	2.05 (1,78; 2.39)	0.0001
**Left Lateral Tumor Extent (cm)**			
Uterine artery	1.84 (1.20;2.27)	1.85 (1.14; 2.06)	0.711
1 cm caudal	1.88 (1.44;2.60)	2.00 (1.39; 2.67)	0.313
2 cm caudal	1.95 (0.18; 2.41)	1.85 (0.64; 2.32)	0.733
Maximum	2.08 (1.90; 2.82)	2.19 (1.73; 3.11)	0.368

**Spatial indices:** The spatial overlap indices depicted a moderate to strong agreement, with a volume ratio of 0.90 (0.79;1.05), DSC of 0.75 (0.68; 0.80), and JI of 0.61(0.52; 0.66). The MHD on the right and left sides were a median of 0.27 cm (0.13; 0.47) and 0.15 cm (0.03; 0.57) respectively.

#### Pre brachytherapy comparison

**Fig. 3:**
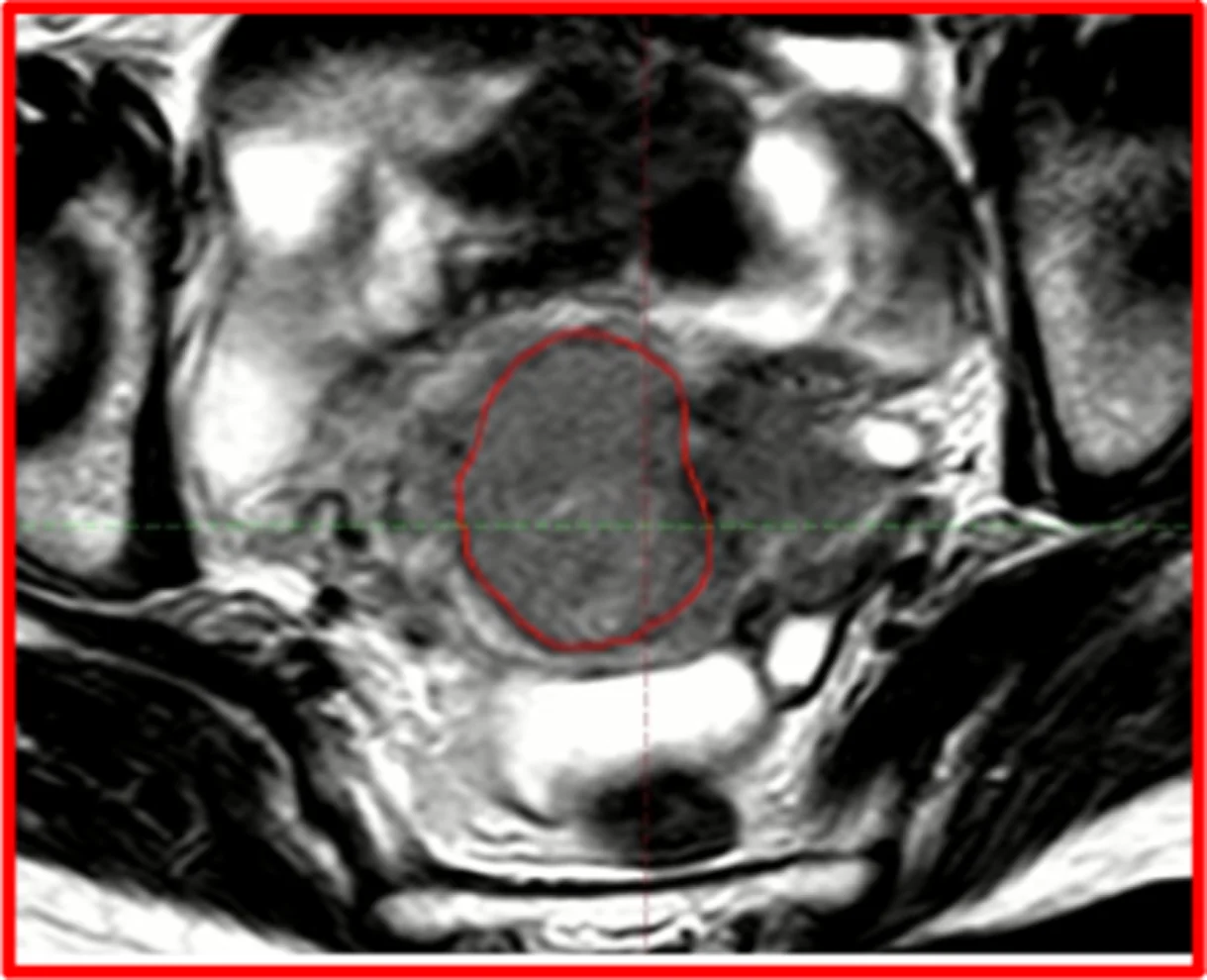

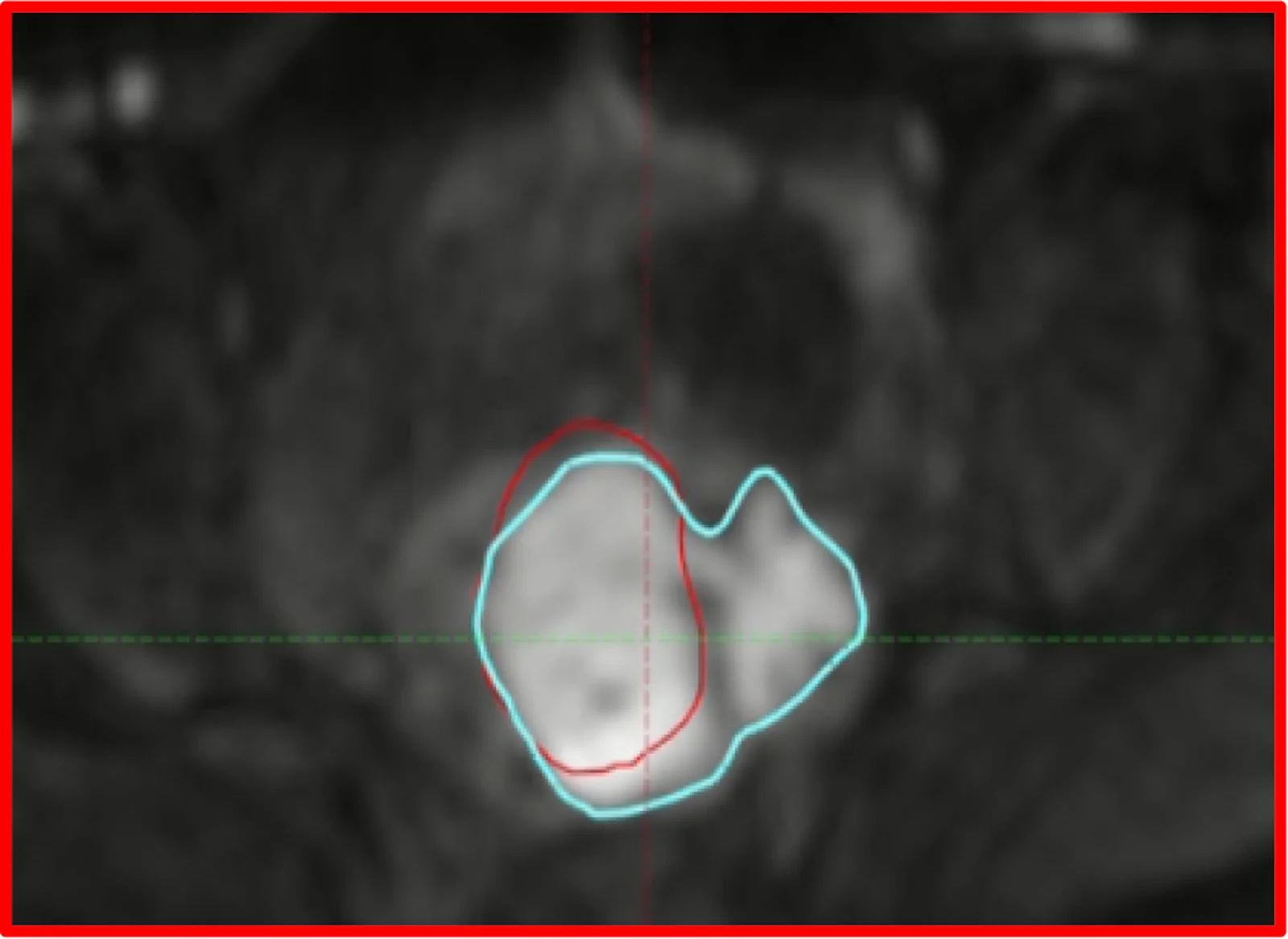
a. Figure depicting the GTV at pre_EBRT, GTV_T2 in red outline with an adjacent hypointense mass in left paracervical region. (For interpretation of the references to colour in this figure legend, the reader is referred to the web version of this article.) b. Figure showing the GTV at pre_EBRT; superimposed GTV_T2 in red outline and GTV_T2/DWI in cyan outline with high diffusion restriction area in left paracervical region corresponding to the hypointense T2 regions. (For interpretation of the references to colour in this figure legend, the reader is referred to the web version of this article.)

**Fig. 4:**
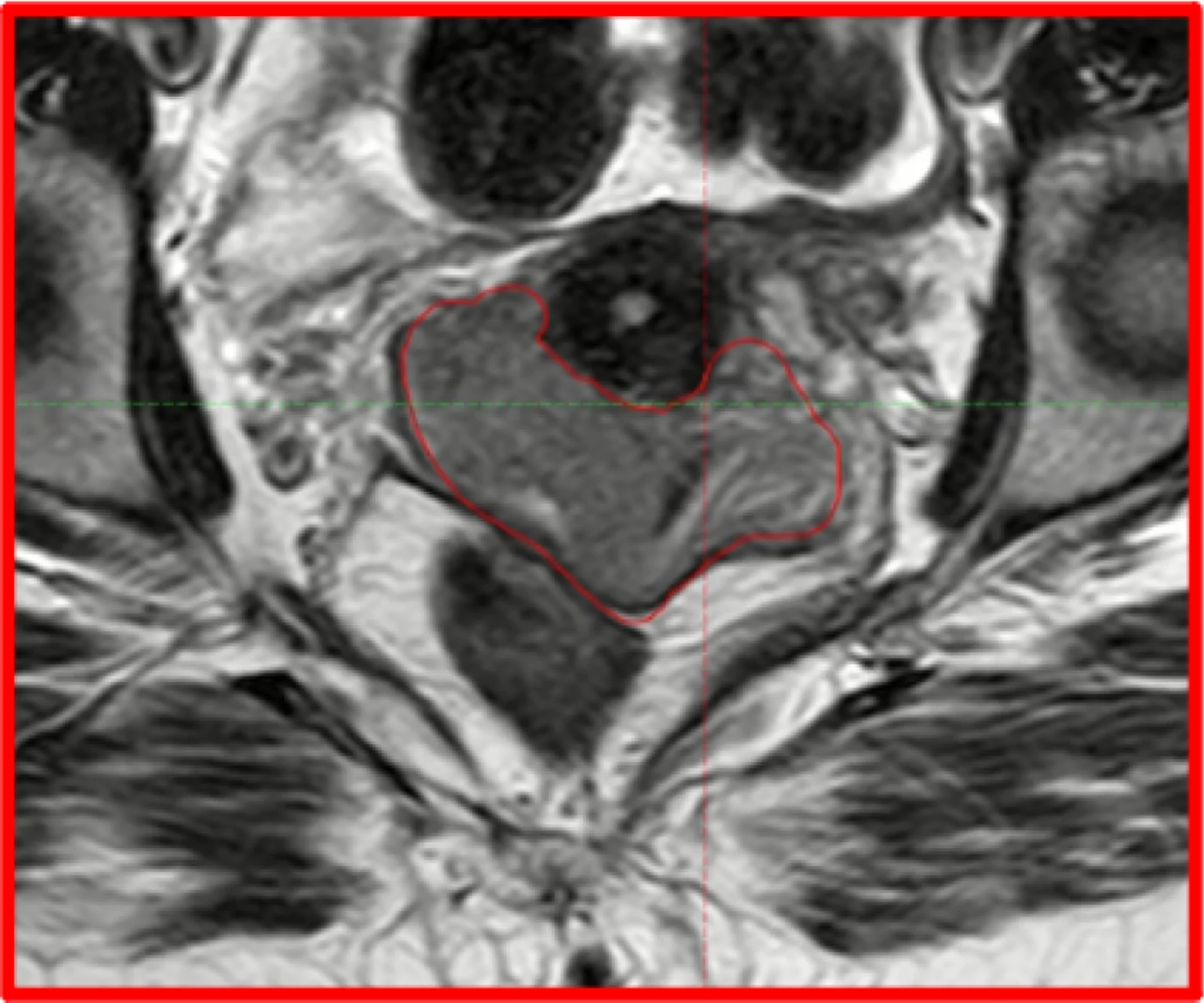

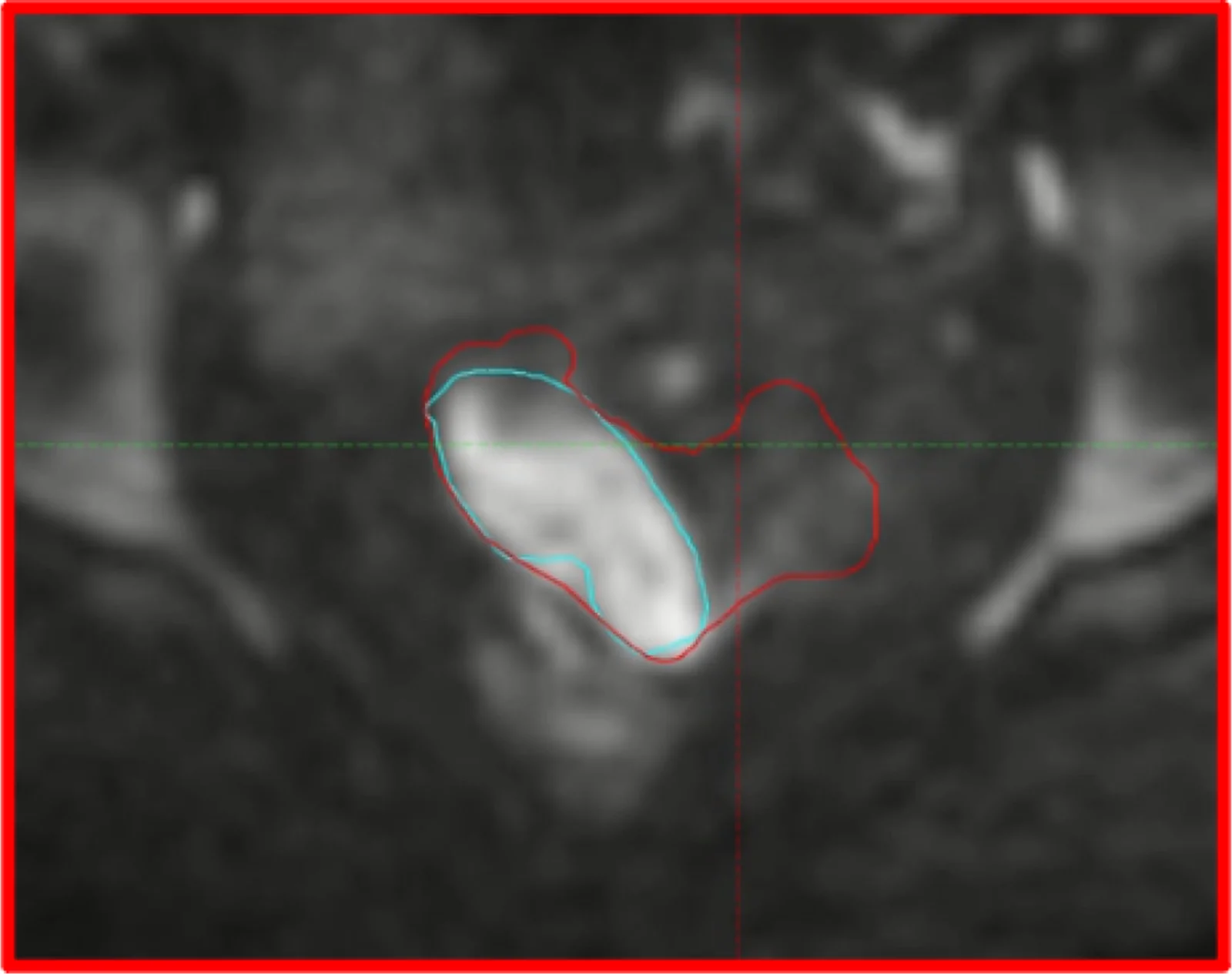
a. Figure showing T2 weighted MRI with GTV_T2 in red outline, depicting bulky tumor at the posterior lip of cervix. (For interpretation of the references to colour in this figure legend, the reader is referred to the web version of this article.) b. Figure showing the GTV at pre_EBRT; superimposed GTV_T2 in red outline and GTV_T2/DWI in cyan outline that depicts no diffusion restriction in the left half. (For interpretation of the references to colour in this figure legend, the reader is referred to the web version of this article.)

**Fig. 5:**
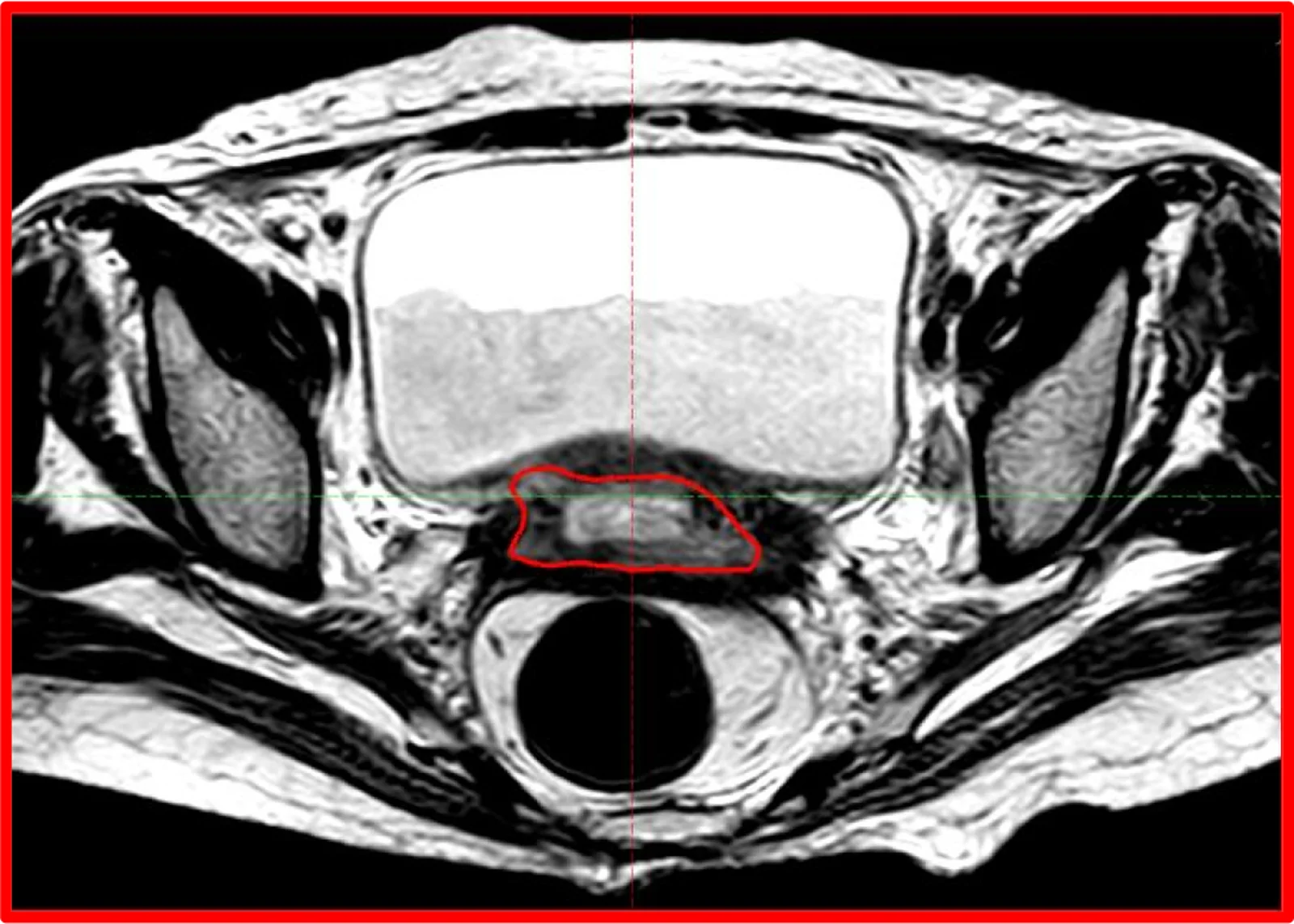

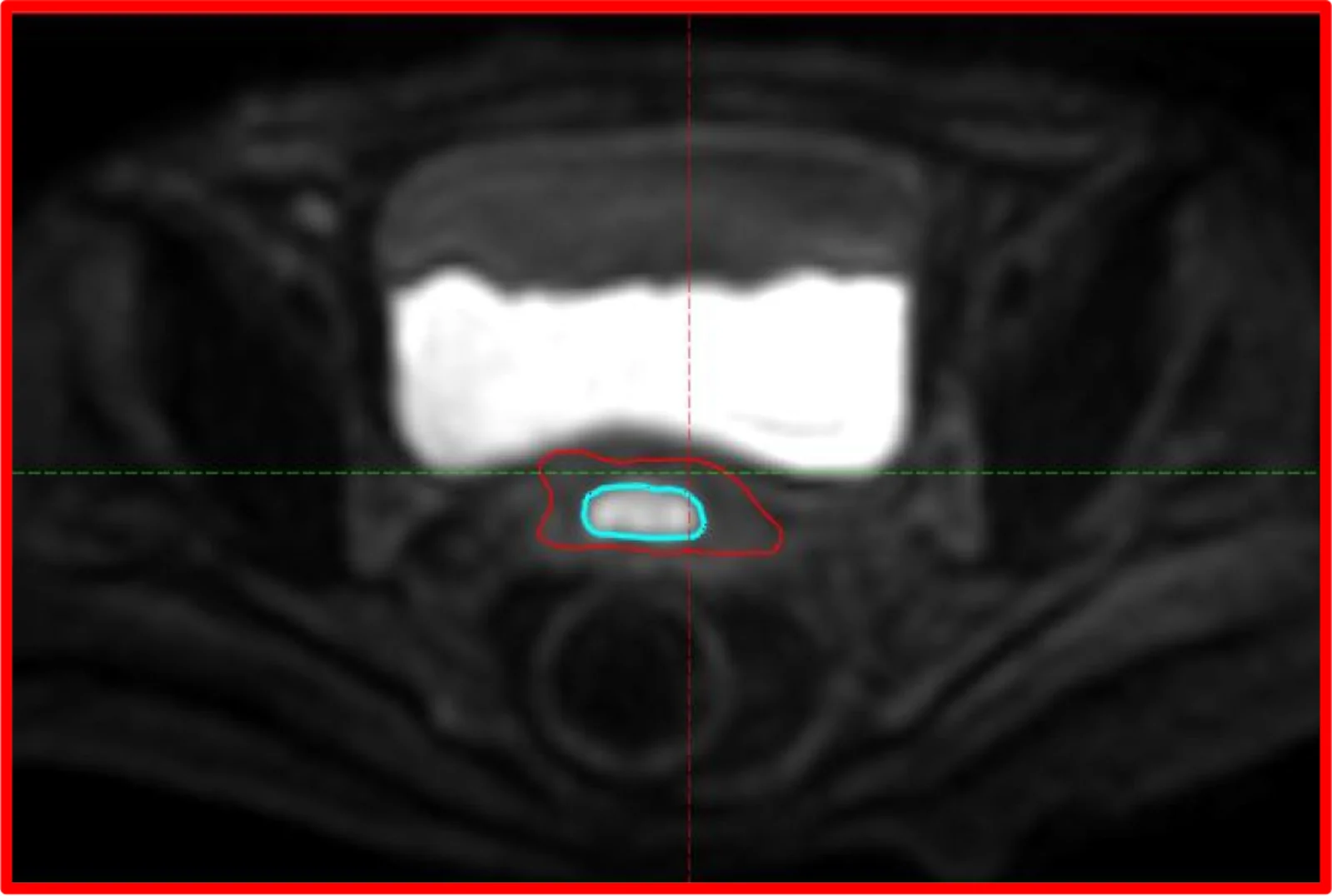
a. Figure showing the GTV at pre_brachytherapy T2 weighted MRI; GTV_T2 in red outline. (For interpretation of the references to colour in this figure legend, the reader is referred to the web version of this article.) b. Figure showing the GTV at pre_brachytherapy on DWI; superimposed GTV_T2 in red outline and GTV_T2/DWI in cyan outline. (For interpretation of the references to colour in this figure legend, the reader is referred to the web version of this article.)

**Fig. 6:**
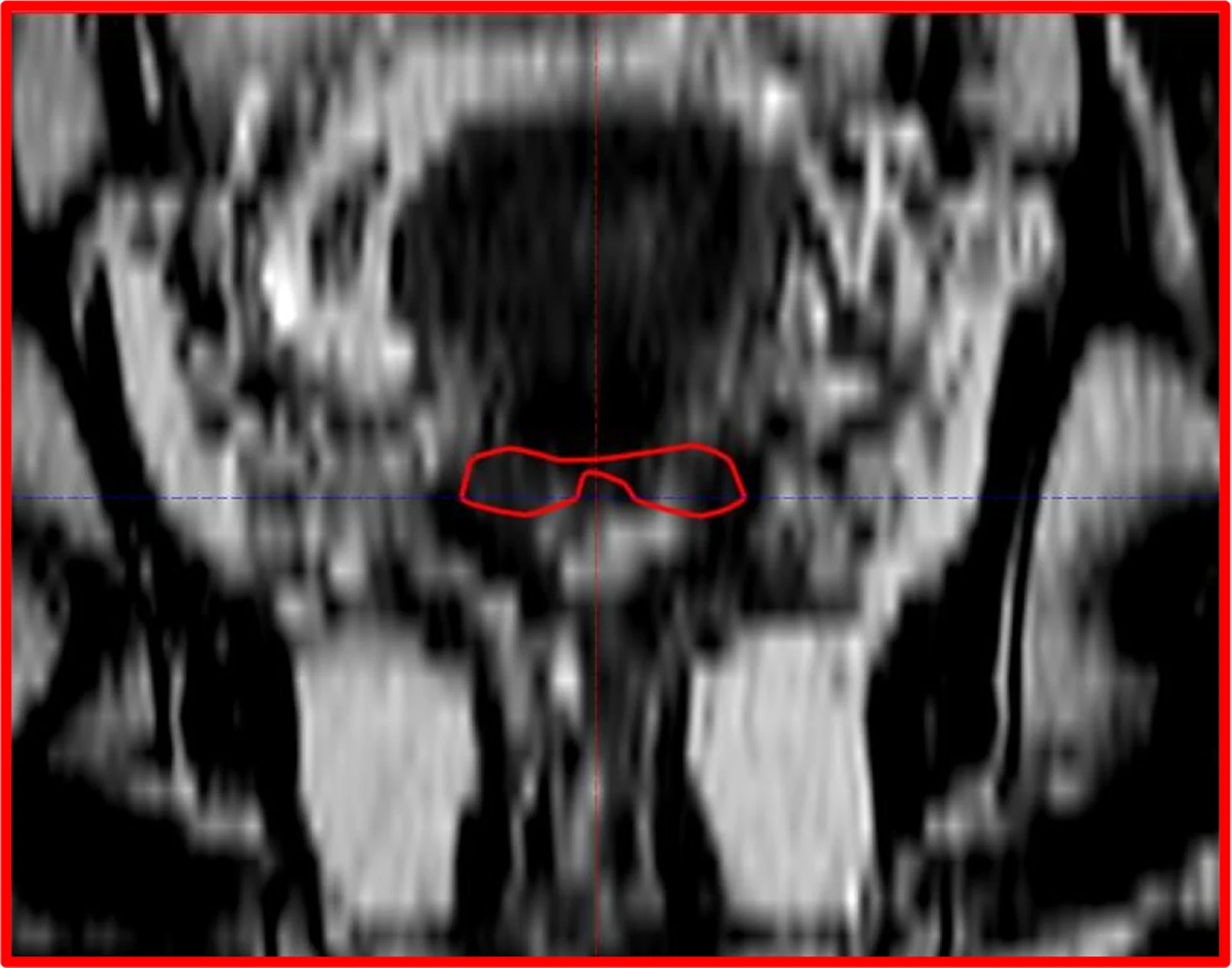

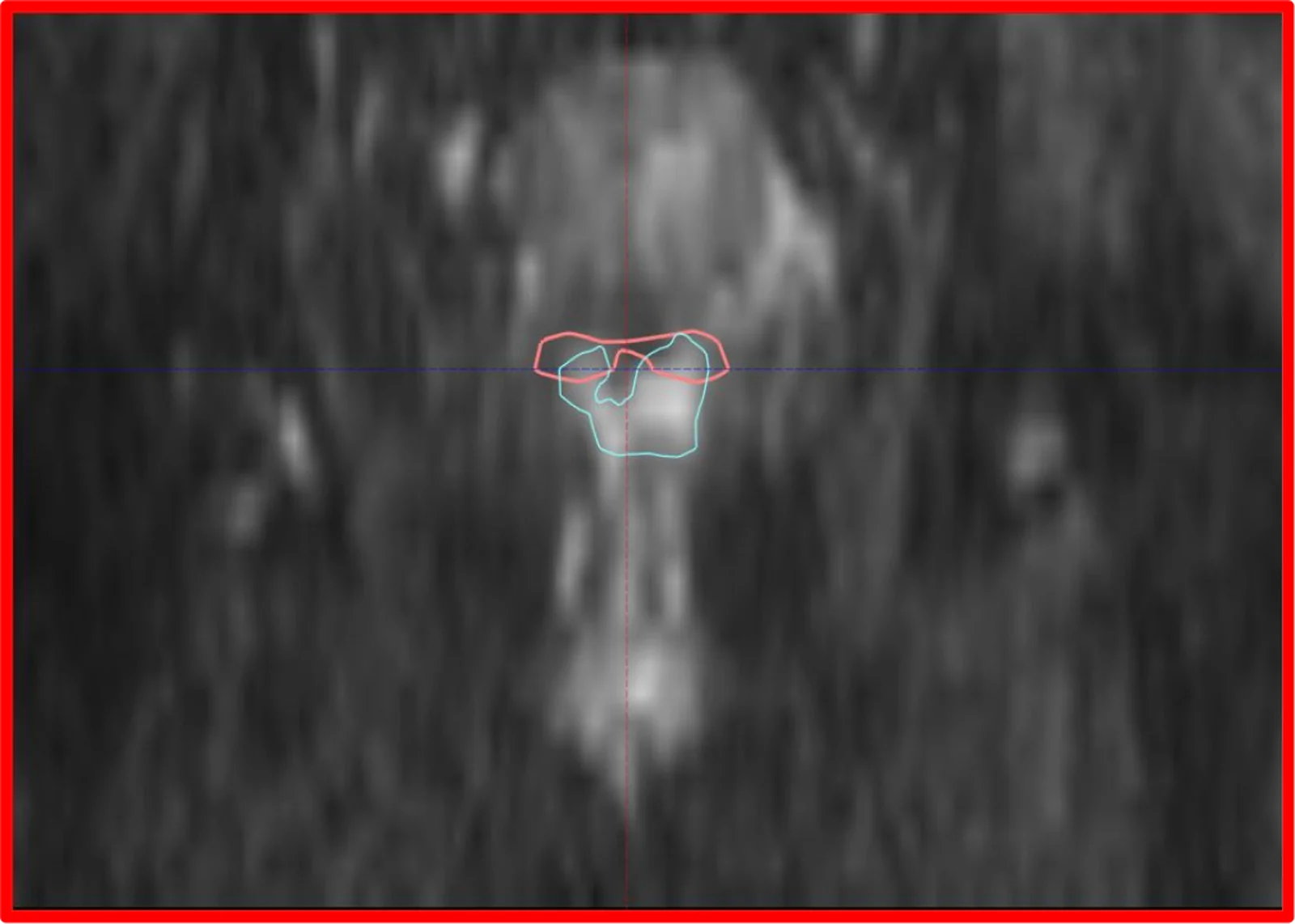

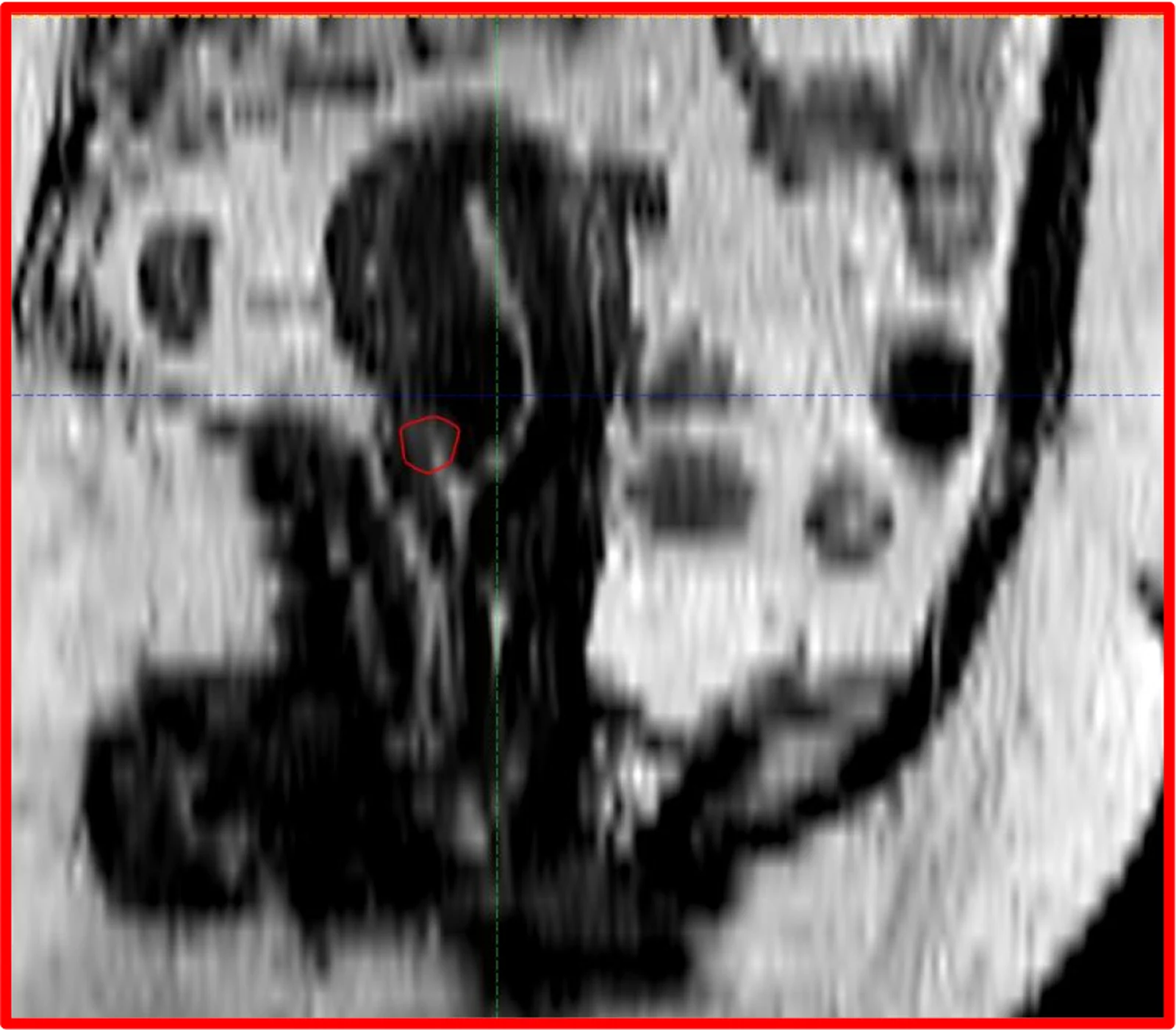

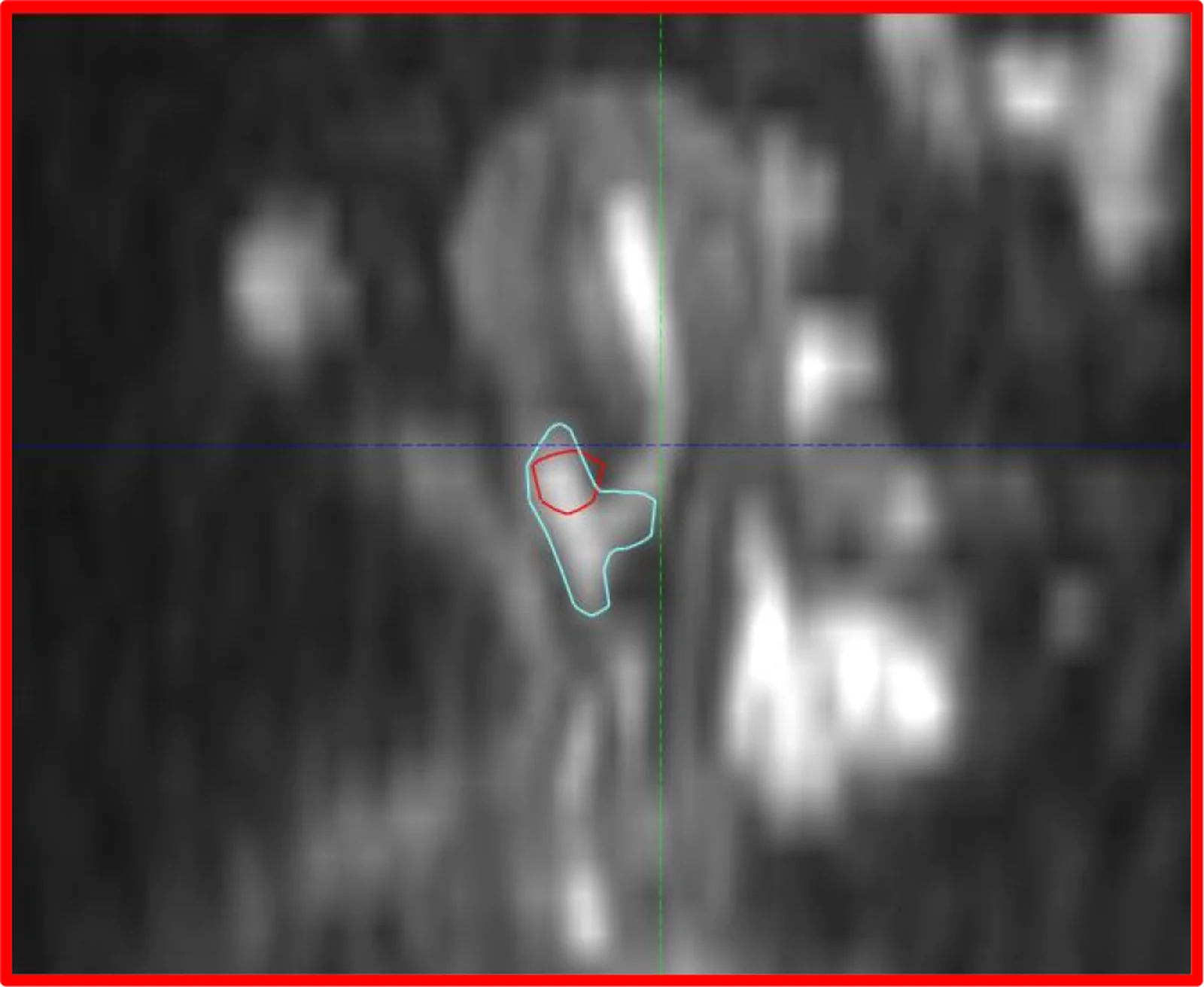
a. Figure showing GTV_T2 at pre-brachytherapy on T2 weighted MRI, coronal view. b. Figures showing GTV_T2/DWI at pre-brachytherapy on DWI in cyan outline with the corresponding GTV_T2 in red outline, coronal view. (For interpretation of the references to colour in this figure legend, the reader is referred to the web version of this article.) c. Figure showing GTV_T2 at pre-brachytherapy on T2 weighted MRI, sagittal view. d. Figures showing GTV_T2/DWI at pre-brachytherapy on DWI in cyan outline with the corresponding GTV_T2 in red outline, sagittal view. (For interpretation of the references to colour in this figure legend, the reader is referred to the web version of this article.)

**Volumetric comparison:** The median difference in absolute volumes was 0.30 cm^3^ (IQR:-0.07; 0.77) with significantly smaller volumes of GTV_T2/DWI volumes compared to GTV_T2, (median 1.05 cm^3^ versus 1.50 cm^3^; p = 0.017). The percentage difference was median of 22.17%, ranging from −58.2% to +200%. The lateral tumor extent measurement at one of the levels was significantly higher on T2 imaging, being 0.95 cm (0.60;1.78) versus 0.81 cm (0.00;1.39) (*p* = 0.033) (Table 3). Out of the 20 patients, 13 exhibited a smaller GTV on the T2/DWI scan; 5 had a larger volume, and the remaining 2 had equal volumes. The two volumes demonstrated a moderately strong positive correlation (ρ = 0.707, *p* = 0.0005). (Table 3) In comparison of the maximum lateral tumor extent of two volumes, majority 29 values, 72.5% were within 5 mm, 7 (17.5%) were ranging from 6 to 10 mm, and 1 value each (total of 10%) fell within range of 11–15 mm, 16–20 mm, 21–25 mm, and > 25 mm. For all the cases, the whole GTV_DWI volume was within the HRCTV. Bland-Altman plots illustrating the differences at pre-brachytherapy are shown in Fig. 2b. Representative examples illustrating discernible differences between imaging modalities at the pre-EBRT and pre-brachytherapy time points are presented in Fig. 3, Fig. 4, Fig. 5, Fig. 6.

**Table 3:** : Table depicting the pre brachytherapy statistical comparison.

Parameter	GTV_T2 Median (IQR)	GTV_T2/DWI Median (IQR)	P value
**Absolute volume (**cm^3^**)**	1.50 (0.80; 3.92)	1.05 (0.60; 3.07)	0.017
**Right Lateral Tumor Extent (cm)**			
Uterine artery	0.00 (0.00; 0.83)	0.00 (0.00; 0.62)	0.123
1 cm caudal	0.73 (0.00; 1.51)	0.71 (0.00; 1.27)	0.456
2 cm caudal	0.00 (0.00; 0.97)	0.00 (0.00; 0.79)	0.237
Maximum	0.95 (0.60; 1.78)	0.81 (0.00; 1.39)	0.033
**Left Lateral Tumor Extent (cm)**			
Uterine artery	0.25 (0.00; 1.39)	0.00 (0.00; 1.04)	0.477
1 cm below	0.77 (0.00; 1.63)	0.85 (0.00; 1.47)	0.507
2 cm below	0.00 (0.00; 1.52)	0.00 (0.00; 1.25)	0.114
Maximum	0.94 (0.60; 1.96)	0.99 (0.30; 1.69)	0.193

**Spatial indices:** The spatial volume overlap indices showed a low to moderate agreement, with a volume ratio of 0.79 (0.48; 1.02), DSC of 0.33 (0.16; 0.56), and JI of 0.19 (0.09; 0.39). There was no significant difference in lateral tumor extent at any of the measured levels. The MHD on the right and left sides were a median of 0.18 cm (0.00; 0.45) and 0.25 cm (0.08; 0.54), respectively. The paired differences of spatial indices (DSC & JI) at pre-EBRT and pre-brachytherapy are depicted in Fig. 2c.

### Exploratory analysis

The absolute tumor volumes quantified by GTV_T2 were not found to be correlated with the percentage differences at brachytherapy, either with pre-EBRT volume (ρ = 0.17, *p* = 0.473); or with pre-brachytherapy volume (ρ = 0.119, *p* = 0.619). Also, no significant correlation was observed between the percentage differences at pre-EBRT and pre-brachytherapy (ρ = 0.008; *p* = 0.975). Among the 12 patients in response category 1, percentage differences ranged from 15.5% to 72.9% (median 27.1%), and in the 8 patients in categories 2/3, the differences ranged from −22.7% to 41.9% (median − 5.8%). Although this effect was not statistically significant (*p* = 0.089), this depicts relatively larger volumes found with DWI/ADC in patients with category 2/3 disease and smaller volumes in category 1 disease. The spatial indices did not demonstrate a consistent directional trend between groups. However, this analysis is largely limited by the small sample size.

## Discussion

In the present study, we observed that integrating functional imaging revealed a distinct tumor topography compared to the standard T2-weighted imaging in a subgroup of patients. In the.

pre-brachytherapy datasets, the difference was more pronounced in terms of a significant and wider percentage difference in volumes, and low to moderate agreement on spatial overlap indices and a significant difference in lateral tumor extent was also found. The low DSC and JI indicate substantial spatial discordance with 65% of cases demonstrated smaller volumes on DWI/ADC, suggesting distinct residual disease topography. The greater variability observed in the pre-brachytherapy datasets can be attributed to EBRT-induced inflammation and fibrosis, which, along with the gross disease, also manifests as abnormal signal intensities on T2-weighted imaging [21]. Comparatively, in pre-EBRT datasets, significant differences in lateral tumor extent values were observed across all three measurements on one side, but the other volumetric comparisons showed no significant variation, and the overlap indices showed better agreement. Nevertheless, the differences in GTV detected with addition of functional imaging can affect the two dose-defining volumes of IGABT, that are the HRCTV and intermediate risk clinical target volume (IRCTV), and given the sharp dose gradients, even subtle volumetric variations and the lateral tumor extents can have potential implications in terms of application choice, and target volume delineation, and planning objectives.

Due to the inherent properties of functional imaging, cellular changes such as membrane disintegration and cell death can be detected depicting viable tumor. DWI captures microstructural alterations through differences in water molecule motion, and ADC maps provide both quantitative and qualitative information for further tissue characterization [2], [22], [23]. Another advantage of DWI is the sharper delineation of tumor boundaries and enhanced visualization of viable tumor regions through hyperintense signals, which may help reduce the delineation uncertainty associated with T2-weighted imaging—a factor that contributes to greater variability in GTV contouring compared with HRCTV and IRCTV [24]. In a recent study of 27 patients, Zimmermann et al. evaluated the impact of incorporating DWI alongside T2-weighted sequences at multiple time points and reported improved tumor visibility, particularly at brachytherapy, as well as enhanced interobserver agreement for both experts and trainees at baseline and pre-brachytherapy imaging [25]. The uncertainties in delineation can impact the dose volume parameters substantially. In the study by Hellebust T et al., the variations in contours led to a substantial difference in the D90 and D100 doses of up to 8–9 Gy for the GTV and 5 Gy for the HRCTV [26]. Hence, given the relatively lesser uncertainties involved, HRCTV is the defined reference volume for dose prescription, but this underscores the need to refine GTV delineation methodology, with functional imaging being a potential tool in this direction.

Functional imaging has an established role as a prognostic marker [22], [27]. A meta-analysis showed a significant correlation of absolute ADC values after the first 3 weeks of radiation, and percentage change with the eventual response [28]. Another prospective study reported that DWI imaging improved specificity for detecting residual tumors to 83–99%, compared with 53–89% for T2-weighted imaging alone [29]. But literature on functional imaging in radiation planning is limited to a very few small-scale series (Table 4). One of the studies by Schernberg A et al. with 44 patients depicted the incremental value of integrating functional imaging. At brachytherapy, the GTV could be delineated in 52% of patients using T2 sequences, which increased to 95% with DWI. Overall, the abnormal signal intensity on DWI pointed out HRCTV modifications in 73% patients, while it was totally encompassed by IRCTV in all patients. However, the D90 doses of the two volumes delineated on T2 and DWI showed no significant difference [16]. In another study by Esthappan J et al., inclusive of 15 patients with a brachytherapy planning MRI, 13 of them had significantly smaller tumor volumes on ADC compared to T2 sequence, with the DSC of 0.54 ± 0.22. Also, the edge contrast based on the pixel values showed a significantly (*p* = 0.001) higher mean percentage difference on ADC (12.7 ± 7.7%) compared to T2 images (4.6 ± 3.2%) [15]. Another study by Kumar R et al. of 50 patients, the GTV volumes on T2 were marginally larger compared to that of DWI, (mean 5.25 cm^3^ versus 5.23 cm^3^; *p* = 0.80). However, the HRCTV volume based on T2 was significantly larger compared to that on DWI (28.4 cm^3^ versus 27.1 cm^3^; *p* = 0.003) with the doses being significantly lesser with T2 based contours. (D90: 7.35 Gy versus 7.41 Gy; *p* = 0.006; D100: 4.41 Gy versus 4.44 Gy; p = 0.006) while OAR's doses showed no significant difference [30].

**Table 4:** Summary of published literature and this study.

Author (Year), Sample Size (N)	MR acquisition timepoint	Absolute volume comparison	Spatial indices	Dosimetric comparison (Gy)
[16] *(2017)* *N* = 44	At brachytherapy, post EBRT	***GTV volume*** (Median, Range) T2: 2.6 cm^3^ (0.4–32.4) DWI: 9.0 cm^3^ (1.3–58.0) Correlation (*r* = 0.58, *P* = 0.038)	**Conformity index** 0.75–1: 7 patients 0.5–0.75: 5 patients	***GTV D 90*** (Median, Range) T2: 37.3 (17.1–48.9) DWI: 33 (22–97) (*P* = 0.659)
[15] (2011) *N* = 15	At brachytherapy, during course of EBRT	***GTV volume*** (Mean ± SD) T2: 64.0 ± 70.5 cm^3^ ADC: 52.3 ± 54.6 cm^3^ *P* = 0.0074	Mean ± SD values ***DSC:*** 0.54 ± 0.22 ≥ 0.7: 5 patients ***Mean percentage edge contrast*** ADC: 12.7 ± 7.7% T2: 4.6 ± 3.2% *P* = 0.001	–
[30] (2020) *N* = 15	At brachytherapy, post EBRT	***GTV volume*** (Mean ± SD) T2: 5.25 ± 2.2 cm^3^ DWI: 5.23 ± 0.8 cm^3^ *P* = 0.8 ***HRCTV volume*** (Mean ± SD) T2: 28.4 ± 5.3 cm^3^ DWI: 27.1 + 3.5 cm^3^ *P* = 0.003	–	Mean ± SD values ***HRCTV*** ***D90:*** T2: 7.35 ± 0.37 DWI: 7.41 ± 0.35 *P* = 0.006 ***D100:*** T2: 4.41 ± 0.22 DWI: 4.44 ± 0.21 P = 0.006 ***Bladder D2cc*** T2: 5.51 ± 0.28 DWI: 5.51 ± 0.27 *P* = 0.9 ***Rectum D2cc*** T2: 4.04 ± 0.21 DWI: 4.04 ± 0.21 P = 0.8 ***Sigmoid D2cc*** T2: 3.31 ± 0.17 DWI: 3.30 ± 0.16 *P* = 0.85
Present Study *N* = 20	At pre EBRT and pre brachytherapy	Pre EBRT: ***GTV volume*** (Median; IQR) T2: 35.25 cm^3^ (11.75;48.42) T2/DWI: 23.75 cm^3^ (12.65; 45.60) *P* = 0.117 Correlation (*r* = 0.943; *P* < 0.0001)	Median (IQR) values ***Volume ratio***: 0.90 (0.79;1.05) ***DSC:*** 0.75 (0.68; 0.80) ***JI:*** 0.61 (0.52; 0.66)	***–***
		Pre brachytherapy: ***GTV volume*** (Median; IQR) T2 = 1.50 cm^3^ (0.80; 3.92) T2/DWI = 1.05 cm^3^ (0.60; 3.07) *P* = 0.017 Correlation (*r* = 0.707; *P* = 0.0005)	***Volume ratio:*** 0.79 (0.48; 1.02) ***DSC:*** 0.33 (0.16; 0.56) ***JI:*** 0.19 (0.09; 0.39)	***–***

*Abbreviations*: SD – Standard Deviation; P - *p* value.

The functional activity of the standard T2-based IGABT volumes was studied by Haack S et al., with quantitative ADC analysis in 15 patients using brachytherapy planning MRI, performed during the last two weeks of EBRT. Mean ADC value for GTV was significantly lower than HRCTV, and that of HRCTV was significantly lower than IRCTV (*p* < 0.001). The GTV comprised 37.2% low-diffusion volume, while the HRCTV and IRCTV contained 20.3% and 10.8%, respectively depicting the prevailing target volumes concept correlation with functional activity [31]. The rationale for the standard T2-based delineation lies in the differential disease burden, as regions with gross disease at baseline that evolve into “grey zones” may harbor a high microscopic disease burden [19]. Also, the current practices are validated by encouraging data from EMBRACE 1 with strikingly high local control rates and minimal morbidity [32]. Recent data on the patterns of local relapses provides additional insights, where 90% of local failures were in-field, of which 50% were inside HRCTV, 15% inside IRCTV and 24% inside both [4]. Given that in-field relapses are predominant, delving into the differential sub volumes and defining the dose prescription to the GTV can possibly enhance the therapeutic window further. The IQ-EMBRACE study aims to determine the prognostic value of multi-parametric MR imaging for relapse patterns [33]. Also, functional imaging can serve as a tool to map the radioresistant necrotic areas, which increases the risk of local recurrence [4].

Our study provides novel insights into the potential of functional imaging beyond volumetric comparisons, through a detailed spatial analysis depicting a distinct disease map in at least a subgroup of patients. Although the impact of functional imaging on target delineation in brachytherapy has been previously reported, our study uniquely integrates the differential impact of timing demonstrating temporal variation with lesser concordance at pre-brachytherapy than pre-EBRT time point. However, at the pre-EBRT time point, we observed striking differences in lateral tumor extent between imaging modalities. To our knowledge, this aspect has not been specifically explored in previous studies and may have important implications for IRCTV delineation, warranting further investigation in future research.

However, there are a few limitations. Firstly, the analysis of ADC was only qualitative and there were inherent limitations of DWI, the T2 shine through effect, motion artefact, pyometra resulting in diffusion restriction, and surrounding air cavities degrading the image quality. Also, the accepted fusion error of up to 2 mm in datasets can influence the comparison of lateral tumor extent measurements and spatial overlap indices, particularly for the small volumes at pre-brachytherapy. These errors are an inherent limitation of multiparametric MRI and techniques including cognitive fusion approach, whereby T2-weighted contours are modified using information from DWI/ADC sequences and deformable registration need to be explored for future research. [34], [35]. A further limitation is that the consensus contours were serially generated for the two volumes that may have introduced potential interpretation bias and precluded formal assessment of inter-observer variability. Nevertheless, our findings provide valid grounds for generating hypotheses to evaluate the potential of functional imaging.

## Conclusion

Integrating functional imaging with standard T2-weighted MRI in cervical cancer can reveal a spatially distinct map for GTV delineation, with differences more pronounced at pre-brachytherapy imaging. This approach requires validation in prospective studies correlating the differential volumes with dose–response relationships and patterns of relapse for refining target volume recommendations.

## CRediT authorship contribution statement

**Ankita Mehta:** Writing – review & editing, Writing – original draft, Supervision, Methodology, Investigation, Formal analysis, Data curation, Conceptualization. **Adarsh Ishwar Hegde:** Writing – original draft, Methodology, Investigation, Formal analysis, Data curation. **Jayashree N P:** Validation. **Umesh Velu:** Validation. **Shirley Lewis:** Writing – review & editing, Writing – original draft, Validation, Supervision, Methodology, Formal analysis, Conceptualization.

## Declaration of competing interest

Dr Shirley Lewis has stock ownership in AIRONC Healthcare Private Limited which is unrelated to current work. Other authors declare that they have no known competing financial interests or personal relationships that could have appeared to influence the work reported in this paper.

## References

[1] Dimopoulos J.C., Petrow P., Tanderup K. (2012). Recommendations from gynaecological (GYN) GEC-ESTRO working group (IV): basic principles and parameters for MR imaging within the frame of image based adaptive cervix cancer brachytherapy. Radiother Oncol.

[2] Hande V., Chopra S., Kalra B. (2022). Point-a vs. volume-based brachytherapy for the treatment of cervix cancer: a meta-analysis. Radiother Oncol.

[3] Sasidharan A., Mahantshetty U., Gurram L. (2020). Patterns of first relapse and outcome in patients with locally advanced cervical cancer after radiochemotherapy: a single institutional experience. Indian J Gynecol Oncol.

[4] Schmid M.P., Lindegaard J.C., Mahantshetty U. (2023). EMBRACE collaborative group. Risk factors for local failure following chemoradiation and magnetic resonance image-guided brachytherapy in locally advanced cervical Cancer: results from the EMBRACE-I study. J Clin Oncol.

[5] Viswanathan A.N., Erickson B., Gaffney D.K. (2014). Comparison and consensus guidelines for delineation of clinical target volume for CT- and MR-based brachytherapy in locally advanced cervical cancer. Int J Radiat Oncol Biol Phys.

[6] Li M., Zhang Q., Yang K. (2021). Role of MRI-based functional imaging in improving the therapeutic index of radiotherapy in cancer treatment. Front Oncol.

[7] Guimaraes M.D., Schuch A., Hochhegger B. (2014). Functional magnetic resonance imaging in oncology: state of the art. Radiol Bras.

[8] Press R.H., Shu H.G., Shim H. (2018). The use of quantitative imaging in radiation oncology: a quantitative imaging network (QIN) perspective. Int J Radiat Oncol Biol Phys.

[9] Padhani A.R. (2011). Diffusion magnetic resonance imaging in cancer patient management. Semin Radiat Oncol.

[10] Koh D.M., Takahara T., Imai Y. (2007). Practical aspects of assessing tumors using clinical diffusion-weighted imaging in the body. Magn Reson Med Sci.

[11] Padhani A.R., Koh D.M. (2011). Diffusion MR imaging for monitoring of treatment response. Magn Reson Imaging Clin N Am.

[12] Pasquini L., Peck K.K., Jenabi M. (2023). Functional MRI in neuro-oncology: state of the art and future directions. Radiology.

[13] Fernandes M.C., Gollub M.J., Brown G. (2022). The importance of MRI for rectal cancer evaluation. Surg Oncol.

[14] Miranda J., Causa Andrieu P., Nincevic J. (2023). Advances in MRI-based assessment of rectal cancer post-neoadjuvant therapy: a comprehensive review. J Clin Med.

[15] Esthappan J., Ma D.J., Narra V.R. (2011). Comparison of apparent diffusion coefficient maps to T2-weighted images for target delineation in cervix cancer brachytherapy. J Contemp Brachyther.

[16] Schernberg A., Balleyguier C., Dumas I. (2017). Diffusion-weighted MRI in image-guided adaptive brachytherapy: tumor delineation feasibility study and comparison with GEC-ESTRO guidelines. Brachytherapy.

[17] Fournier L.S., Bats A.S., Durdux C. (2020). Diffusion MRI: technical principles and application to uterine cervical cancer. Cancer Radiother.

[18] Pötter R., Tanderup K., Kirisits C., EMBRACE Collaborative Group (2018). The EMBRACE II study: the outcome and prospect of two decades of evolution within the GEC-ESTRO GYN working group and the EMBRACE studies. Clin Transl Radiat Oncol.

[19] International commission on radiation units and measurements (2016).

[20] Mahantshetty U., Poetter R., Beriwal S. (2021). IBS-GEC ESTRO-ABS recommendations for CT based contouring in image guided adaptive brachytherapy for cervical cancer. Radiother Oncol.

[21] Vincens E., Balleyguier C., Rey A. (2008). Accuracy of magnetic resonance imaging in predicting residual disease in patients treated for stage IB2/II cervical carcinoma with chemoradiation therapy: correlation of radiologic findings with surgicopathologic results. Cancer.

[22] Abdul-Latif M., Tharmalingam H., Tsang Y. (2023). Functional magnetic resonance imaging in cervical Cancer diagnosis and treatment. Clin Oncol (R Coll Radiol).

[23] Matani H., Patel A.K., Horne Z.D. (2022). Utilization of functional MRI in the diagnosis and management of cervical cancer. Front Oncol.

[24] Petrič P., Hudej R., Rogelj P. (2013). Uncertainties of target volume delineation in MRI guided adaptive brachytherapy of cervix cancer: a multi-institutional study. Radiother Oncol.

[25] Zimmermann L., Knäusl B., Knoth J., Sturdza A., Dick V., Mrva-Ghukasyan T. (2026). Diffusion weighted imaging for gross tumor volume delineation in primary radiochemotherapy and image guided adaptive brachytherapy for cervical cancer. Radiother Oncol.

[26] Hellebust T.P., Tanderup K., Lervåg C. (2013). Dosimetric impact of interobserver variability in MRI-based delineation for cervical cancer brachytherapy. Radiother Oncol.

[27] McVeigh P.Z., Syed A.M., Milosevic M. (2008). Diffusion-weighted MRI in cervical cancer. Eur Radiol.

[28] Harry V.N., Persad S., Bassaw B. (2021). Diffusion-weighted MRI to detect early response to chemoradiation in cervical cancer: a systematic review and meta-analysis. Gynecol Oncol Rep.

[29] Thomeer M.G., Vandecaveye V., Braun L. (2019). Evaluation of T2-W MR imaging and diffusion-weighted imaging for the early post-treatment local response assessment of patients treated conservatively for cervical cancer: a multicentre study. Eur Radiol.

[30] Kumar R., Narayanan G.S., Vishwanthan B., Narayanan S., Mandal S. (2020). A prospective comparative dosimetric study between diffusion weighted MRI (DWI) & T2-weighted MRI (T2W) for target delineation and planning in cervical cancer brachytherapy. Rep Pract Oncol Radiother.

[31] Haack S., Pedersen E.M., Jespersen S.N. (2010). Apparent diffusion coefficients in GEC ESTRO target volumes for image guided adaptive brachytherapy of locally advanced cervical cancer. Acta Oncol.

[32] Pötter R., Tanderup K., Schmid M.P. (2021). EMBRACE collaborative group. MRI-guided adaptive brachytherapy in locally advanced cervical cancer (EMBRACE-I): a multicentre prospective cohort study. Lancet Oncol.

[33] (2017). IQ-EMBRACE Quantitative MR Imaging in Locally Advanced Cervical Cancer Sub-study under the EMBRACE II protocol.

[34] Tait L.M., Hoffman D., Benedict S. (2016). The use of MRI deformable image registration for CT-based brachytherapy in locally advanced cervical cancer. Brachytherapy.

[35] Cao X., Yang J., Gao Y. (2018). Region-adaptive deformable registration of CT/MRI pelvic images via learning-based image synthesis. IEEE Trans Image Process.

